# Differential temporal activation of oxy- and deoxy-hemodynamic signals in optical imaging using functional near-infrared spectroscopy (fNIRS)

**DOI:** 10.1186/1471-2202-16-S1-P245

**Published:** 2015-12-18

**Authors:** Nicoladie D Tam, George Zouridakis

**Affiliations:** 1 Department of Biological Sciences, University of North Texas, Denton, TX 76203, USA; 2 Departments of Engineering Technology, Computer Science, and Electrical and Computer Engineering, University of Houston, Houston, TX, 77204, USA

## Background

Optical imaging of the brain based on near-infrared spectroscopy (NIRS) can provide real-time measurements of the hemodynamic signals that represent metabolic demands of the underlying neural tissues. Functional imaging based on NIRS (fNIRS) can detect both oxy-hemoglobin (oxy-Hb) and deoxy-hemoglobin (deoxy-Hb) levels related to neural metabolic activity, whereas BOLD fMRI (blood-oxygen-level dependent functional magnetic resonance imaging) can only detect signals related to deoxy-Hb. Thus, during task execution, only fNIRS can determine the differential temporal activation/deactivation of oxy-Hb and deoxy-Hb hemodynamic signals as the blood-oxygen demand changes. We have previously shown that as metabolic demand increases, temporal changes in oxy-Hb and deoxy-Hb levels can be temporally *decoupled *(i.e., oxy-Hb level can decrease while deoxy-Hb level increases) rather than being coupled, in which case both would increase or decrease simultaneously [1-5]. In order to account for the observed differential temporal decoupling of oxy-Hb and deoxy-Hb levels, we hypothesize that as oxygen demand increases, the delivery of blood oxygen cannot keep up with the demand of the neural tissues, resulting in decreased oxy-Hb and increased deoxy-Hb levels. This study provides experimental evidence that validates the above hypothesis.

## Methods:

Human subjects were recruited to execute voluntary arm movements in orthogonal directions to exert different oxygen demands onto the motor cortex. The hemodynamic activities were recorded from the motor cortex using fNIRS, while the subjects executed predefined arm movements. The oxy-Hb and deoxy-Hb levels were computed from the NIRS optical signals using the modified Beer-Lambert law [6].

## Results:

**Figure 1:**
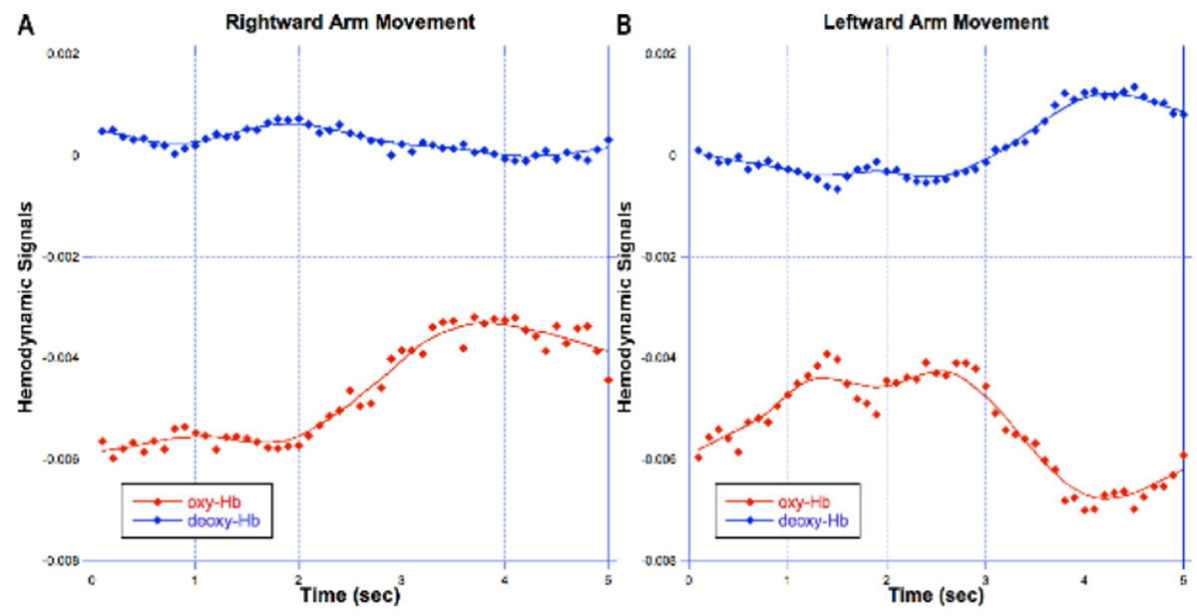
**Optical hemodynamic signals recorded from the motor cortex, showing different oxygen demands**. (A) Rightward arm movement. (B) Leftward arm movement. [Oxy-Hb (in red) and deoxy-Hb (in blue)]

Figure 1 shows the differential changes of oxy- and deoxy-Hb hemodynamic signals over time during right and left movement directions. These data demonstrate that the oxy- and deoxy-Hb hemodynamic signals can change differentially rather than being coupled in time. The differential changes in oxy- and deoxy-Hb levels can be accounted by an oxygen demand exceeding the oxygen delivery in the blood vessels.

## Conclusions:

The metabolic demands of the neural tissues are not necessarily correlated with either oxy- or deoxy-Hb alone, but they are correlated with the combination of both oxy- and deoxy-Hb. A decrease in oxy-Hb level does not necessarily imply that oxygen demand decreases. Rather, such a decrease in oxy-Hb level can be due to the rate of oxygen demand by the neural tissues that exceeds the oxygen delivery capacity of the blood vessels.
